# Eco-Friendly Chitosan Production by *Syncephalastrum racemosum* and Application to the Removal of Acid Orange 7 (AO7) from Wastewaters

**DOI:** 10.3390/molecules18077646

**Published:** 2013-07-01

**Authors:** Anabelle C. L. Batista, Marta C. Freitas Silva, Jefferson B. Batista, Aline Elesbão Nascimento, Galba M. Campos-Takaki

**Affiliations:** 1 Rede Nordeste de Biotecnologia (RENORBIO), Universidade Federal Rural de Pernambuco, Rua Dom Manoel de Medeiros, s/n, Dois Irmãos—52171-900 Recife, PE, Brasil; E-Mail: abatista@ufersa.edu.br; 2 Departamento de Ciências Animais (DCAN), Universidade Federal Rural do Semi-Árido, Av. Francisco Mota, 572—Costa e Silva—59625-900 Mossoró, RN, Brasil; 3 Núcleo de Pesquisas em Ciências Ambientais (NPCIAMB), Universidade Católica de Pernambuco, Boa Vista 50 050-590 Recife, PE, Brasil; E-Mails: martacfs@yahoo.com.br (M.C.F.S.); elesbao@unicap.br (A.E.N.); 4 Instituto Federal de Educação, Ciência e Tecnologia da Paraíba, Av. 1° de Maio, Jaguaribe 58015-430 João Pessoa, PB, Brasil; E-Mail: biojef13@yahoo.com.br

**Keywords:** microbiological chitosan, acid orange 7 (AO7), central composite rotational design, agro-industrial residues, *Syncephalastrum racemosum*, corn steep liquor, coagulation-flocculation

## Abstract

Due to the existence of new methodologies that have reduced the production costs of microbiological chitosan, this paper puts forward the use of agro-industrial residues in order to produce microbiological chitosan and to apply chitosan as an innovative resource for removing acid orange 7 (AO7) from wastewaters. The best culture conditions were selected by a full 2^4^ factorial design, and the removal of the dye was optimized by a 2^3^ central composite rotational design. The results showed that corn steep liquor (CSL) is an agro-industrial residue that can be advantageously used to produce microbiological chitosan with yields up to 7.8 g/kg of substrate. FT-IR spectra of the product showed typical peak distributions like those of standard chitosan which confirmed the extracted product was chitosan-like. The efficiency of removing low concentrations of AO7 by using microbiological chitosan in distilled water (up to 89.96%) and tap water (up to 80.60%) was significantly higher than the efficiency of the control (chitosan obtained from crustaceans), suggesting that this biopolymer is a better economic alternative for discoloring wastewater where a low concentration of the dye is considered toxic. The high percentage recovery of AO7 from the microbiological chitosan particles used favors this biopolymer as a possible bleaching agent which may be reusable.

## 1. Introduction

Among the polymers most investigated today, there is chitosan, a polysaccharide formed by glycosidic amino residues of β-(1-4)-2-amino-2-deoxy-d-glucopyranose, which is obtained by deacetylation of α- or β-chitin found mainly in the shells of crustaceans and mollusks, respectively [1], or by the specific action of the enzyme chitin deacetylase (EC 3.5.1.41) on the residues of γ-chitin present in the cell walls of fungi [2,3,4]. Among the chitosan-producing fungi, emphasis is given to the class Zygomycetes [4,5,6,7,8,9,10], as the species *Syncephalastrum racemosum* has great potential for producing chitosan in low cost culture media [11].

Currently, chitosan is produced from α-chitin, because the production values are commercially advantageous. However, obtaining chitosan from α-chitin requires some care regarding standardizing the product [12,13] and incorporating this biopolymer into production lines of different biotechnological areas. In addition, this method of obtaining chitosan has adverse environmental implications as it produces millions of gallons of acidic and basic residues, which are then discharged into the environment, usually without treatment and without a view to re-use [14].

Given this situation, obtaining chitosan by submerged culture of fungi has the advantage of manipulating and standardizing specific physicochemical characteristics, thus facilitating its incorporation into industrial production lines, besides helping to reduce the environmental waste generated when producing chitosan from the deacetylation of α-chitin [11,15,16]. Nowadays, the acquisition of chitosan from fungal strains has emphasized the use of industrial wastes as an alternative nutritional source for obtaining a byproduct of high value added [6,7,11], and it has been claimed that this leads to a decrease of 38 to 73% in the total production costs [17].

The great interest in producing chitosan is justified by its potential in biotechnological applications, especially with regard to using it in medical and environmental areas [18,19,20,21,22]. In the environmental area, especially regarding the removal of dye from textile effluents, chitosan has proved that it can remove a high amount of azo dyes [19,23,24,25] in particular by the method of coagulation-flocculation, where the chitosan acts as a polyelectrolyte which has no potential environmental polluter. Despite the efficiency of this method, it is important to emphasize that the treatment of wastewater tends to be most effective, easy and inexpensive if carried out on the site of the plant site that is producing pollutants, because, after reaching the effluent, the dye can interact with other molecules in the environment and this results in expensive procedures for identifying and removing specific dyes.

With the increase in textile consumption and interest in diversifying colors, industries have increased the use of color additives in the production of clothing, for example acid orange 7 or Orange II (AO7). AO7 is a synthetic acid dye that is toxic at a concentration of 0.011 mg/mL as expressed by the acute toxicity test EC_50_.

Thus, due to the need to reduce the costs of production and enhance the efficiency of fungi in producing chitosan, this paper assesses the interactive influence of different factors (nutritional source, initial pH of culture, temperature of incubation and inoculum size) by a full factorial design and applying chitosan as a coagulant agent of azo dye acid orange 7. Despite there already being extensive knowledge of the mechanisms of interactions between chitosan and azo dyes, this study innovates by applying a 2^3^ Central Composite Rotational Design (CCRD), with four central points, to evaluate the simultaneous effect of chitosan concentration, dye concentration and pH on effluent decolorization by coagulation-flocculation.

## 2. Results and Discussion

### 2.1. Effects on Chitosan Production

The simplest and most effective statistical method for analyzing different factors that can interfere in the response to a product is a full factorial design [26], since this enables the possible main effects for the factors analyzed in a specific situation to be estimated. In this study, the nutritional source, the initial pH and the temperature of incubation were considered as important factors for reducing the production costs and enhancing the productivity of chitosan. Corroborating the studies by Bartnicki-Garcia and Nickerson [27], the size of the inoculum did not influence the amount of microbiological chitosan produced, independent of the influence of the variables studied.

In our study, the CSL which was used as the sole source of carbon and nitrogen in batch positively influenced the microbiological production of chitosan. This fact may have occurred due to high concentrations of nitrogen (17.57%) and carbohydrate (13.03%) found in the CSL concentrate used in this research. The results corroborate those in the literature, which also describe the use of CSL as a good source of nitrogen for the production of biopolymers [7,17,28]. The discrepancy between the values of the nutritional compositions of the CSL concentrate used may have occurred due to the origin of the maize.

The experimental design used to analyze the main effects between the independent factors (the concentration of CSL, initial pH, temperature and size of the inoculum) on the microbiological production of chitosan showed that the best assay occurred when the dilution of concentrated CSL was only 2% (v/v), with initial pH 8.0, 25 °C and an inoculum size of 10^2^ spores/mL. For these conditions, chitosan was produced at 62.44 mg per gram of dry biomass, or 7.8 g of chitosan per kilogram of CSL concentrate. This result was superior to the results of Wang *et al.* [7], who obtained a yield of 6.12 g of chitosan per kilogram of substrate concentrate (CSL + molasses) using *Absidia coerulea*. This fact corroborates the literature which describes *S. racemosum* as a good producer of chitosan in different nutritional media [11]. 

As described by Aranaz *et al.* [29], the applicability of chitosan is dependent on its physico-chemical properties. For this reason, analyses were performed to characterize the chitosans, and showed a deacetylation degree (DD) = 88.14% for microbiological chitosan and DD = 78.54% for standard chitosan. A high degree of deacetylation indicates a large amount of free amino groups present in the molecule, which, in acidic solution, are protonated and may form favorable electrostatic interactions with other molecules, an example of which is the SO^3−^ groups of the structure of azo dyes [30,31,32].

In studies of crystallinity, microbiological chitosan showed a high crystallinity (crystallinity index = 55.96%) and standard chitosan was seen to be amorphous, suggesting a lower amount of intermolecular structural links between the amino groups of residues in the standard chitosan when compared with microbiological chitosan [29,33]. During the process of transforming α-chitin into chitosan, some very careful steps must be taken so as not to degrade the polymer chain, thus generating an amorphous chitosan [34,35]. This does not happen when chitosan is formed by the specific action of chitin deacetylase under the γ-chitin in fungi. The high degree of crystallinity is also described as favoring the adsorption of acid dyes by chitosan [36].

In the analysis of viscosimetric molecular weight, the microbiological chitosan showed a low molecular weight, while standard chitosan showed an average viscosimetric molecular weight. The importance of molecular weight on coagulation-flocculation process is noted in the literature which states that the lower molecular weight of chitosan is related to the increased efficiency in removing azo dyes in systems where the solvent has a low ionic strength (distilled water) [31].

After selecting the culture medium for the low-cost production of chitosan, production was scaled up to obtain satisfactory amounts of chitosan to be used to evaluate its application in removing AO7, which was used in this study as a dye model for treating from textile effluents .

### 2.2. Initial Considerations

The literature describes the use of chitosan obtained from the exoskeleton of crustaceans, as an alternative to the chemical treatment of wastewater, and shows that some factors such as the concentration of chitosan in solution, the type of chemical pollutant, pH, the type and concentration of dye and ionic strength of the solution are significant variables in the adsorption process by chitosan [19,23,24,25,37,38]. In this paper, the importance of microbiological chitosan as an alternative substance for discoloring textile wastewater was first tested by CCRD (see Table 1). 

The coagulant-flocculant action, at first, was influenced by the origin and physico-chemical characteristics of chitosan and by the ionic strength of the water used as solvent (Table 1), and obtained up to 89.91% and 73.54% decolorization efficiency for the microbiological and standard chitosans, respectively. In order to analyse the isothermal equilibrium, the literature suggests using the Langmuir model to describe the adsorption of the dye by chitosan, indicating that the crosslink preferably occurs through the formation of a monolayer on the surface of particles of chitosan [23,25,36,39].

**Table 1:** Results of 2^3^central complete rotational design (CCRD), with six axial points and four central points which show the efficiency in removing the orange acid 7 (AO7) from solutions by microbiological or standard chitosan (CS) in distilled or tap water. Data acquired after 2 h of incubation.

Assay	Independent variables			Discoloration			
	[CS]	[dye]	pH	Microbiological CS		Standard CS	
				Distilled water ^†‡^	Tap water ^‡^	Distilled water ^‡^	Tap water ^‡^
*1*	−1	−1	−1	70.91a	58.21	56.95	49.58
*2*	1	−1	−1	75.45b	61.21	45.54	51.58
*3*	−1	1	−1	34.90c	**80.32**j	68.21	71.53
*4*	1	1	−1	76.62b	73.39	67.57	68.50
*5*	−1	−1	1	55.31d	58.36	56.43	45.42
*6*	1	−1	1	58.63d	61.1	52.37	53.21
*7*	−1	1	1	33.35c	70.69	63.39	**72.03**l
*8*	1	1	1	**89.91**e	72.66	67.68	71.68
*9*	−1.68	0	0	14.91f	32.23	**72.84**l	71.79
*10*	1.68	0	0	66.18g	68.23	62.17	67.03
*11*	0	−1.68	0	71.27a	52.88	38.46	40.04
*12*	0	1.68	0	56.67d	**80.6**j	64.88	**73.57**l
*13*	0	0	−1.68	85.28h	72.48	68.48	67.21
*14*	0	0	1.68	80.57i	66.27	63.87	55.53
*15* *	0	0	0	75.26bi	62.20	62.94	65.12

* Results expressed as the average of quadruplicates; ^†^ Values in the same column with the same letter are not significantly different (*p* < 0.05) when Tukey's HSD tests are used; ^‡^ Values underlined, in different columns, and with the same letter are not significantly different (*p* < 0.05) when Tukey’s HSD tests are used.

### 2.3. Factorial Analysis

#### 2.3.1. Effect of the Origin and Physicochemical Characteristics of Chitosan on the Coagulation-Flocculation of AO7

**Figure 1:**
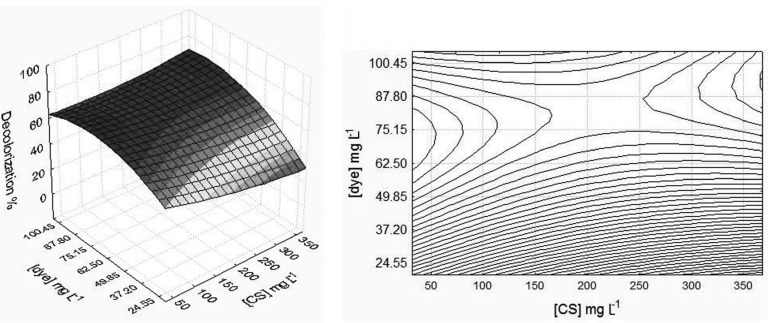
The 3D-surface plot (**left**) and 2D-projection (**right**) showing the interactions between standard chitosan concentration and dye concentration at pH 3.0 on discoloration efficiency in distilled water.

In the study of the main effects of the chitosan standard (Sigma), it was observed that for the systems where the solvent was distilled water there was a significant result (*p* < 0.05) for the factors of chitosan concentration and dye concentration, which enabled the variance analysis and showed the efficiency of the decolorization by using the response surface methodology (Figure 1). The result was not significant for the range of pH, for the parameters considered, which may be for the reasons suggested by Wang *et al.* [7] and Focher *et al.* [33]: most of the amino groups of the amorphous chitosan are shown to be protonated, independently of the pH in solution.

Figure 1 shows that the efficiency in removing AO7 was greater when there was a low concentration of standard chitosan and in the range of 60–80 mg/L of dye. It is suggested that the amorphous condition of standard chitosan may have favored accessibility to the amino sites of the polymer [7,29,31,33] up to a limit, when the concentration of dye saturated the system and there was a need to increase the concentration of chitosan so that the efficiency of removing AO7 from the solution was further improved.

**Figure 2:**
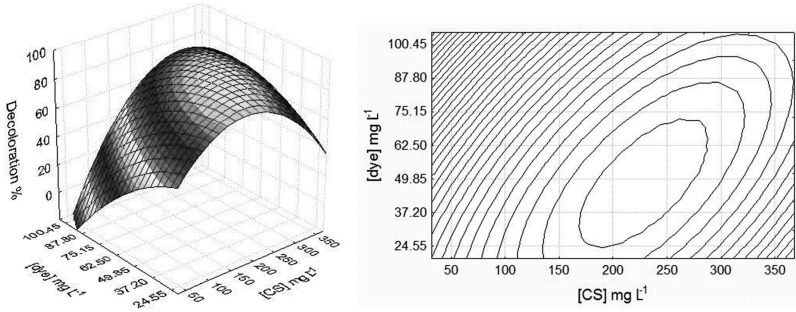
The 3D-surface plot (**left**) and 2D-projection (**right**) showing the interactions between microbiological chitosan concentration and dye concentration at pH 3.0 on decoloration efficiency in distilled water.

In systems where microbiological chitosan was added in distilled water, only the concentration factors of chitosan and the concentration of dye were significant (*p* < 0.01) for representation in a quadratic model. Using the response surface generated by the model (Figure 2), one can obtain the conditions of chitosan and dye concentration that resulted in a greater removal of AO7 from the solution, for which an optimum range of 180–280 mg/L was observed for the concentration of chitosan and 24.55 to 73.58 mg/L for the concentration of dye. The significance of the result for microbiological chitosan, when compared to the removal efficiency of standard chitosan in distilled water, can be observed by means of the Tukey test (Table 1). The greater efficiency in removing AO7, up to 89.91%, by microbiological chitosan in comparison with standard chitosan is suggested by the higher degree of deacetylation (DD) and the high crystallinity shown by microbiological chitosan [31], which corroborates data given by Wong *et al.* [36], who state that an amorphous condition of chitosan reduces the adsorption capacity for acid dyes. Another possible influence on the greater efficiency in removing the dye by microbiological chitosan its lower molecular weight compared with standard chitosan [31,40]. 

**Figure 3:**
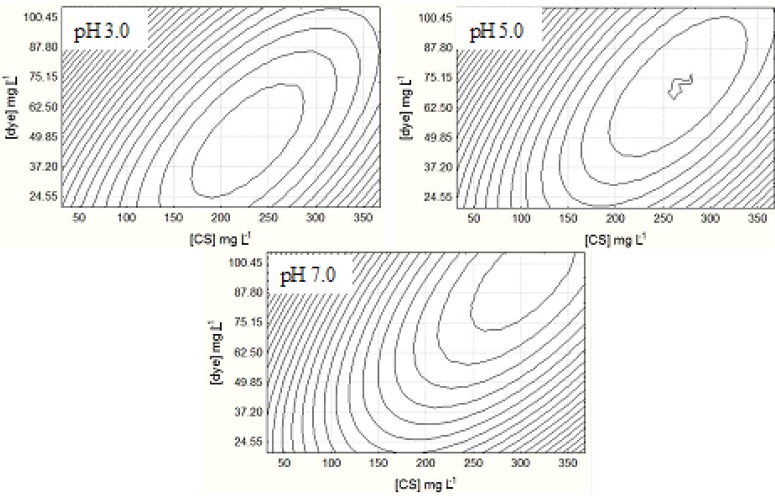
The 2D-projections showing the interactions between microbiological chitosan concentration and dye concentration at different pH evaluated by CCRD in distilled water.

Although, no significant main effect was observed for the factor pH in systems where the solvent was distilled water, what can be stressed is that there is a linearity between the amount of microbiological chitosan required to be bound to the dye and the pH value of the solution (Figure 3). This fact can be explained by the solvent properties of the chitosan, which, in acid pH, presents mostly NH^3−^ reactive sites, thus enabling the formation of electrostatic bonds in a larger amount than in systems where the pH is closer to the neutral value [7,31,32]. Szygula *et al.* [19] also observed that the lower the pH value the less the amount of chitosan that is required to bind with the reactive sites of the azo dye AB92 (acid blue 92).

To analyze the effects of the ionic strength of the solvent on the systems where microbiological or standard chitosan were added, a complete factorial design was carried out both on distilled water and tap water. 

In the assays where microbiological chitosan was applied, significant differences (*p* < 0.05) were observed when the ionic strengths of the solvent were compared on the removal of AO7, as observed by the Tukey test (Table 1). It is suggested that the high degree of deacetylation and low molecular weight of microbiological chitosan were important characteristics for the greater efficiency in removing AO7 in distilled water [31], since the ionic strength of the solvent may have interfered with the sorption mechanism between the protonated amino groups of chitosan and the anions of the dye [30,32]. 

In the tests for applying standard chitosan, it can be observed from the Tukey test that there were no significant differences dependent on the ionic strength of the solvent, and this suggests that the amorphous condition of the molecule of the standard chitosan has favored the formation of electrostatic bonds between the chitosan and AO7 regardless of the ionic strength. For systems where standard or microbiological chitosan was applied, in tap water, the only significant effect was for the dye concentration factor, thus making it impossible to apply this factor to equational modeling so as to assess the main effects or interactions. 

#### 2.3.2. Analysis of the Effects of the Ionic Strength of the Solvent in Removing AO7 by Chitosan

The difference from the influence of the tap water used in the study by Szygula *et al.* [19] is suggested, mainly, on account of the difference in the ion composition observed for the two kinds of water, particularly because of the amount of sulfate ions (SO^−2^), which was suggested as influential in the process of removing the azo dye Acid Blue 92 (AB92). However, the tap water used in this experiment had a low sulfate concentration (data not shown), which can be differentiated in the results obtained. The physico-chemical properties of the chitosans are another factor suggested as important for explaining the difference in the results.

Although a higher removal efficiency of AO7, up to 89.91%, was observed due to the action of microbiological chitosan in distilled water, the high cost of using distilled water at industrial levels suggests the use of microbiological chitosan in systems where the solvent may have to be tap water. The suggestion arises from the fact that regardless of the ionic strength of the solvent, microbiological chitosan showed higher efficiency in removing AO7, when compared with standard chitosan (chitosan obtained from crustaceans) (Table 1).

#### 2.3.3. Modeling for the Removal Percentage of AO7 by Microbiological Chitosan in Solvent of Low Ionic Strength

The general quadratic model was used to explain the higher removal percentage of AO7 by microbiological chitosan in a solution of low ionic strength (distilled water) for the range of values selected in this study:

Y = β0 + ∑βjxj + ∑βixixj + ∑βjjxj2

where Y is the predicted response, β0 is the offset term, βi is the linear displacement, is the square βii is the quadratic offset, βij is the effect of the interaction, i < j, and xi is the non-dimensional coded value of Xi [41].

In Table 1, assay 8 showed a significantly higher decolorization percentage value, up to 89.91%, when compared to other conditions. Response Y predictions for the percentage of decolorization for assay 8 was obtained from Equation (1).

Ydecol. (%) = 78.36 + 14.09 × 1 − 3.66 × 2 − 2.09 × 3 − 13.31 × 12 − 5.01 × 22 + 11.30 × 12 +5.52 × 23
(1)

The statistical significance of the prediction response equation was certified by the F test and by variance analysis (ANOVA). The ANOVA (data not shown) of the quadratic regression model demonstrated that the model was significant, having a value *p* < 0.01 and R^2^ = 0.983, thus demonstrating the applicability of the model when the experiment is projected.

### 2.4. Recovery of the AO7 Dye Using the Flocculant Agent

The recovery of the dye from the particles of microbiological and standard chitosan used during the experiments, in both distilled or tap water, was performed by solubilizing the flocculant agent in 30 mL of 0.1 M NaOH per sample of the agent obtained after carrying out each assay [19]. From this analysis it was possible to recover and concentrate in volume ten times smaller, approximately 95% of the dye used, regardless of type of chitosan or of the ionic strength of the solvent. Similar results were obtained by Szygula *et al.* [19] where approximately 100% of the azo dye bound to chitosan, obtained from crustacean, was recovered. Studies on reusing chitosan (microbiological or standard) dissociated from the dye were not possible due to the small amount used in CCRD. It is suggested that future studies consider conducting tests for re-microbiological chitosan with a view to reducing the cost of the process for removing azo dye from solutions.

## 3. Experimental

### 3.1. Microorganism and Culture Media

Sub-cultures of *Syncephalastrum racemosum* (WFCC/UCP-0148), grown on Potato Dextrose Agar (PDA, Oxoid, Kansas city, USA) at 28 °C for 120 h were stored and used to make the spore solutions. For biomass production, *S. racemosum* was grown in an aerated environment (150 rpm) for 120 h. The culture media were formulated from corn steep liquor (CSL) as the sole source of carbon and nitrogen, in accordance with full 2^4^ factorial designs with three central points (Table 2). For the formulation of culture media a concentrate of CSL was diluted in distilled water (v/v) in the proportions described in Table 1. Fermentation was performed in a 1,000 mL Erlenmeyer flask, containing 400 mL of culture medium. The chitosan produced was extracted according to Hu *et al.* [42]. After selecting the best production condition by full factorial design, the fermentation processes were scaled to a 2,800 mL Erlenmeyer flask, containing 1,200 mL of culture medium. Full factorial design was performed in duplicate and analyzed using the Statistica 7.0 software [43]. The statistical significance of the results was tested at *p* < 0.05 level.

**Table 2:** 2^4^ Full factorial design, with three central points. Coded (CD) and uncoded (UCD) values to assess the main effects of independent variables on chitosan production.

Independent Variables	CD (UCD)	CD (UCD)	CD (UCD)
CSL	−1 (2%)	0 (6%)	1 (10%)
pH	−1 (4.0)	0 (6.0)	1 (8.0)
Temperature	−1 (25 °C)	0 (31 °C)	1 (37 °C)
Size of inoculums	−1 (10^2^ spores/mL)	0 (5 × 10^5^ spores/mL)	1 (10^6^ spores/mL)

CSL = corn steep liquor.

### 3.2. Characterization of Corn Steep Liquor and Chitosan

To conduct a percentage analysis of total carbohydrate and total nitrogen we used the methodologies described by Cunniff [44] and Kjeldahl [45], respectively.

The degree of deacetylation (DD) of chitosan was determined by infrared spectroscopy and calculated by Equation 2 [46]. The crystallinity index was calculated as per Equation (3) [33]:

DD (%) = [100(Abs1655/Abs3450)]/1.33
(2)

Crystallinity index (%) = 100{[I(θc) − I(θa)]/I(θc)}
(3)

where I (θc) is the relative intensity of the crystalline (2θ = 20°) and I (θa) corresponds to amorphous regions (2θ = 12°) for chitosan.

The molecular weight of chitosan was estimated by viscometry as per the methodology proposed by Terbojevich and Cosani [47]. The measurement was made from the average of the solutions of chitosan (0.3–0.6) in 1% acetic acid, always carried out in triplicate. For this procedure, an Ubbelohde viscometer (Model B806 0C, Cannon Instrument Company, State College, PA, USA) was used. 

### 3.3. Azo Dye Solutions

The azo dye, acid orange 7 (C.I. 15510; molecular weight 350.33 g/mol), also known as Orange II, is a monoazo compound with a reactive sulfonic group and negative loads in aqueous solution (Figure 1a). It was obtained from Sigma-Aldrich Corporation (St. Louis, MO, USA) and used without purification. To standardize the experiments, an acid orange 7 (AO7) stock solution was made (5 mg mL^−1^) and then diluted (v/v) as per Table 3.

**Table 3:** Central composite rotational design (CCRD), 2^3^ with six axial points and four central points was used to analyse the flocculant-coagulant action of chitosan (CS). The same CCRD was used to assess the action of microbiological and standard chitosan in distilled water or tap water. Coded (CD) and uncoded (UCD) values are given in the Table.

Factors	CD (UCD) –α	CD (UCD)	CD (UCD)	CD (UCD)	CD (UCD) +α
[CS]	−1.68 (32 mg/mL)	−1 (100 mg/mL)	0 (200 mg/mL)	1 (300 mg/mL)	1.68 (368 mg/mL)
[dye]	−1.68 (20 mg/mL)	−1 (37 mg/mL)	0 (62.5 mg/mL)	1 (88 mg/mL)	1.68 (105 mg/mL)
pH	−1.68 (1.6)	−1 (3.0)	0 (5.0)	1 (7.0)	1.68 (8.3)

Factors = Independent variables.

### 3.4. Chitosan Solutions

**Figure 4:**
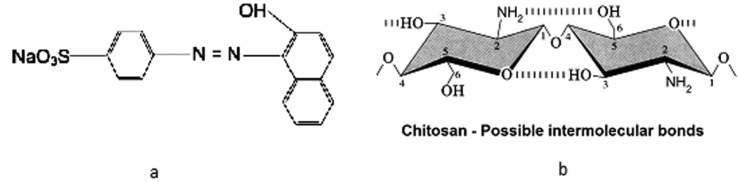
(**a**) Chemical structure of azo dye acid Orange 7 (AO7); (**b**) Chemical structure of chitosan and possible intermolecular bonds.

Commercial chitosan, described as chitosan obtained by alpha-chitin, was used during the experiments as a comparative standard (Sigma-Aldrich) and dissolved in acetic acid without purification. The microbiological chitosan used in the coagulation-flocculation experiments was obtained from *S. racemosum* grown in the best condition selected by the full factorial design described in Table 2 and used without purification. The chitosans (Figure 4b) were ground and sieved, after which 1 mm size fractions were collected and used to prepare the coagulant solutions. The stock solutions of coagulant (1% w/v) were dissolved in 1% acetic acid (v/v) and then stored for use in all experiments.

### 3.5. Coagulation-Flocculation Experiments

To assess the main effects and interactions between the independent factors (chitosan concentration, dye concentration and initial pH) in the process of coagulation-flocculation of the dye AO7 by standard and microbiological chitosan, a 2^3^ Central Composite Rotational Design (CCRD), with six axial points and four central points (Table 3) was proposed. The interference factors were determined in accordance with the literature [18,19,24]. Cestari *et al.* [24] described the temperature factor as statistically not interfering with the mechanisms of coagulation-flocculation by chitosan. The experiments were performed in random order so as to evaluate the experimental errors properly [48].

Homogeneous solutions of the dye were separated into beakers, each containing 300 mL. The pH of each solution was measured and adjusted to a fixed value. After such adjustment, different volumes of chitosan solution (1% v/v) were added (depending on the condition described in Table 3).

The experiments of coagulation-flocculation of the dye by chitosan were performed as per Roussy *et al.* [49] and Szygula *et al.* [19] with some modifications. The experiments were performed at room temperature (26 °C ± 1.0 °C). The samples collected were centrifuged at 12,240 × g for 20 min and filtered. The amount of dye retained on the membrane was negligible (less than 1.5%).

### 3.6. Decolorization Assay

The collected samples were filtered and read upon dye absorption (485 nm) using a Genesis Spectronic UV-vis spectrophotometer, model 2 (Spectronic Instruments Inc., San Francisco, CA, USA). The percentage of decolorization was calculated by Equation (4):

Decolorization (%) = 100((T0 − T2H)/T0)
(4)

where T0 is the dye concentration in the initial phase of the experiment and T2H is the dye concentration after 2 h of the experiment. The amount of dye removed from the solution was assumed as the total bound to the total quantity of chitosan present in the mixture.

## 4. Conclusions

In this study the tested concentrations of Orange AO7 ranged from 20 to 105 mg/mL. The concentrations chosen for the tests were intended to demonstrate the ability of chitosan, a non-toxic polymer that degrades easily in the environment, as a substitute for expensive processes that are potentially toxic to aquatic organisms present in the environment. Based on the results obtained, it is noticed that chitosan was capable of removing up to 80.32% of dye present in tap water in situations where a concentration of 88 mg/mL is dependent on the conditions tested in only 2 h of incubation. Additional analyses associated with the use of “acetosyringone” laccase to oxidize low concentrations of AO7 (17.52 mg/L) at non-toxic levels have been described in the literature [50], but the efficiency procedures using microorganisms for removing azo dye are better at low concentrations of AO7 [51], thus demonstrating the need to pre-treat water contaminated with microbiological azo dye using chitosan to reduce levels of dye in the water to levels that are non-toxic to microorganisms.

The high efficiency removal of different concentrations of AO7 tested by microbiological chitosan rather than crustacean chitosan suggests this microbiological polymer is an economic and eco-friendly alternative that can be applied to the discoloration of wastewater. The low cost of producing high quality microbiological chitosan by *Syncephalastrum racemosum* in wastewater from industrial food industries further reduces the cost of removing AO7 from wastewater from the dyeing industry.

Despite the fact the removal of AO7 by microbiological chitosan in distilled water compared with the efficiency in tap water was more efficient, the high cost to industry of using distilled water suggests the use of microbiological chitosan in systems where tap water is a solvent. The high percentage recovery of AO7 from the microbiological chitosan particles used during the CCRD experiments favors the reuse of this biopolymer.
